# Detection of coronary artery disease at 3 Tesla using a visual interpretation algorithm combining perfusion and delayed enhancement imaging

**DOI:** 10.1186/1532-429X-11-S1-P47

**Published:** 2009-01-28

**Authors:** Jayanth R Arnold, Theodoros D Karamitsos, Jane M Francis, Tammy J Pegg, Nick Searle, Stefan Neubauer, Joseph B Selvanayagam

**Affiliations:** John Radcliffe Hospital, Oxford, UK

**Keywords:** Cardiovascular Magnetic Resonance, Significant Coronary Artery Disease, Myocardial Stress Perfusion Imaging, Reversible Perfusion Defect, Combine Algorithm

## Introduction

Myocardial stress perfusion imaging with cardiovascular magnetic resonance (CMR) is increasingly used in the assessment of coronary artery disease (CAD). It has been demonstrated that a multiparametric approach combining perfusion and infarction imaging at 1.5 Tesla further augments the diagnostic performance of CMR. Recent studies indicate that 3 Tesla is the preferred field strength for perfusion imaging, with increased signal-to-noise and contrast-to-noise ratios compared with 1.5 Tesla. We sought to assess the diagnostic performance of a visual interpretation algorithm combining perfusion and infarction imaging at 3 Tesla.

## Methods

Subjects scheduled for elective diagnostic angiography for investigation of exertional chest pain were studied prior to angiography. Patients were studied with first-pass perfusion at 3 Tesla (Trio, Siemens Medical Solutions), at stress (140 mcg/kg/min intravenous adenosine) and at rest. Three short-axis images were acquired every heartbeat using a saturation recovery fast gradient echo sequence and 0.05 mmol/kg contrast agent (Gadodiamide, Omniscan™, GE Healthcare) bolus injection. Perfusion images were acquired every cardiac cycle during the first pass of contrast, using a T_1_-weighted fast gradient echo sequence (echo time 1.04 ms, repetition time 2 ms, voxel size 2.1 × 2.6 × 8 mm^3^). After rest perfusion, following a further bolus of Gadodiamide (0.045 mmol/kg), delayed enhancement CMR was performed with a T1-weighted segmented inversion-recovery turbo fast low-angle shot (FLASH) sequence (echo time 4.8 ms, voxel size 1.4 × 2.4 × 8 mm^3^, flip angle 20°). Resting cine, stress and rest perfusion, and delayed enhancement images were interpreted visually by a single observer blinded to clinical and angiographic data. The diagnosis of CAD was determined by the presence of delayed hyperenhancement or reversible perfusion defects. Matched stress-rest perfusion defects in the absence of delayed enhancement were considered artifactual. To determine interobserver variability, all scans were interpreted by a second blinded observer. Quantitative coronary angiography, performed by a third operator blinded to CMR results, served as the reference standard. Significant CAD was defined angiographically as the presence of >= 1 stenosis of >= 50% diameter in any of the main epicardial coronary arteries or their branches with a diameter of >= 2 mm.

## Results

**Figure 1:**
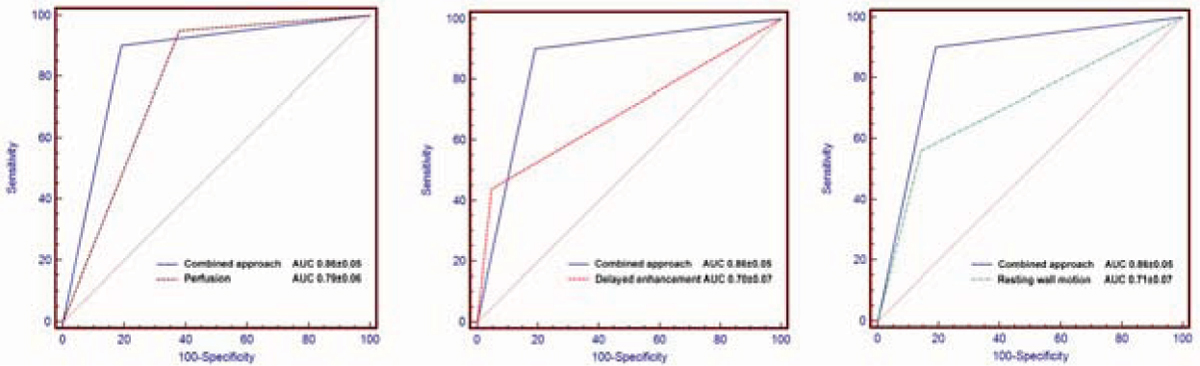
**ROC curves comparing the combined visual interpretation algorithm and individual components for the detection of significant CAD**.

Sixty-five subjects were prospectively recruited. Two individuals did not complete the CMR examination owing to claustrophobia and one withdrew consent, so 62 subjects were included in the final analysis. The prevalence of CAD was 66%. All CMR images were visually interpretable. For the diagnosis of significant CAD, the combination of perfusion and delayed enhancement CMR had a sensitivity, specificity and diagnostic accuracy of 90%, 81%, and 87% respectively, compared with 95%, 62%, and 84% for perfusion alone. The combined algorithm had higher specificity and accuracy owing to the high specificity (95%) of delayed enhancement imaging. The addition of resting wall motion analysis did not improve the diagnostic yield further. Receiver operating characteristic curve analysis demonstrated that the combination of perfusion and delayed enhancement imaging provided better performance than perfusion, delayed enhancement or wall motion assessment alone (figure 1). Diagnostic accuracy was high in single-vessel and multivessel disease (84% and 81%, respectively), and independent of CAD location (LAD 82%, LCx 81% and RCA 81%). There was excellent interobserver agreement for the overall determination of CAD using the combined algorithm (kappa = 0.82).

## Conclusion

CMR imaging at 3 Tesla has high diagnostic accuracy for the identification of significant CAD. Combining perfusion and infarction imaging is superior to perfusion imaging alone and further augments diagnostic performance.

